# Prediction of blood supply in vestibular schwannomas using radiomics machine learning classifiers

**DOI:** 10.1038/s41598-021-97865-5

**Published:** 2021-09-23

**Authors:** Dixiang Song, Yixuan Zhai, Xiaogang Tao, Chao Zhao, Minkai Wang, Xinting Wei

**Affiliations:** Department of Neurosurgery, The First Affiliated Hospital of Zhengzhou University, Zhengzhou, Henan People’s Republic of China

**Keywords:** CNS cancer, Surgical oncology

## Abstract

This study attempts to explore the radiomics-based features of multi-parametric magnetic resonance imaging (MRI) and construct a machine-learning model to predict the blood supply in vestibular schwannoma preoperatively. By retrospectively collecting the preoperative MRI data of patients with vestibular schwannoma, patients were divided into poor and rich blood supply groups according to the intraoperative recording. Patients were divided into training and test cohorts (2:1), randomly. Stable features were retained by intra-group correlation coefficients (ICCs). Four feature selection methods and four classification methods were evaluated to construct favorable radiomics classifiers. The mean area under the curve (AUC) obtained in the test set for different combinations of feature selecting methods and classifiers was calculated separately to compare the performance of the models. Obtain and compare the best combination results with the performance of differentiation through visual observation in clinical diagnosis. 191 patients were included in this study. 3918 stable features were extracted from each patient. Least absolute shrinkage and selection operator (LASSO) and logistic regression model was selected as the optimal combinations after comparing the AUC calculated by models, which predicted the blood supply of vestibular schwannoma by K-Fold cross-validation method with a mean AUC = 0.88 and F1-score = 0.83. Radiomics machine-learning classifiers can accurately predict the blood supply of vestibular schwannoma by preoperative MRI data.

## Introduction

Vestibular schwannomas (VS) or acoustic neuroma are benign nerve sheath tumors arising from Schwann cells of the acoustic nerve, comprise about 80% of cerebellopontine angle (CPA) tumors^1–3^. The annual incidence of VS is 0.6–0.8 per 100,000 people^4^. The blood supply of VS is an important factor affecting the difficulty of surgery. Previous studies have reported on Hypervascular Vestibular Schwannomas^5–8^, and several studies have shown that an abundant blood supply is significantly leading to an occurrence of facial impairment^9^ and decrease in rate of resection^8^. The current method of preoperative assessment of the blood supply in VS is mainly by visual identification of the anatomical presentation of MRI and the degree of signal enhancement on T1-CE sequences. Empirically, the higher signal of the tumor on T1-CE sequences, the finding of multiple flow voids on the tumor surface or in the tumor parenchyma, and solid tumors with less cystic^5,6,8^ suggest a rich blood supply to the tumor. In clinical practice, however, this method usually does not provide a satisfactory accuracy rate. Radiomics is considered to be a promising method of analyzing medical images^10^, which can be combined with machine-learning methods to guide the clinic by building predictive models. The main purpose of this study is to mine the radiomics features from preoperative multi-parametric MRI of patients with VS and build a machine-learning model to predict the blood supply of VS. In this study, we extracted a large number of features from the MRI data of 191 cases of patients with VS by radiomics methods, retained the features through consistency testing and feature selection to build machine-learning classifiers for the blood supply of VS, and evaluated the accuracy of the classifiers by cross-validation.

## Clinical materials and methods

### Patients

This study was conducted following approval by the Research Ethics Board, A database of 191 patients with histologically proven primary VS in the First Affiliated Hospital of Zhengzhou University was retrospectively retrieved from December 2013 to August 2020. Approval for the study was obtained from the research ethics committee of the First Affiliated Hospital of Zhengzhou University, and informed consent was obtained from all subjects or, if subjects are under 18, from a parent and/or legal guardian. All methods were performed in accordance with the relevant guidelines and regulations. All patients underwent a preoperative plain and gadolinium-enhanced MRI in our center, the MRI sequences were collected including T1-weighted images, T2-weighted images, T2-weighted-Fluid-Attenuated Inversion Recovery (T2-FLAIR) images, and T1-weighted gadolinium-enhanced (T1-CE) images. Tumors at Stage I (tumors confined to the internal auditory canal, diameter 1–10 mm) by Koos classification^11^ were excluded because the blood supply of such small tumors may be difficult to define through the evaluation criteria of this study, and there was a possibility that insufficient radiomics information could be extracted. All surgical procedures were performed by the same highly qualified neurosurgeon. Patients were placed in a lateral position under general anesthesia during the whole procedure. All procedures were performed via a suboccipital retrosigmoid approach, and after revealing the sigmoid sinus and transverse sinus, the cerebrospinal fluid was fully released and the tumor was resected in pieces under the microscope. The blood supply of the tumor was recorded and confirmed by the senior neurosurgeon and the assistant together. Those who had less bleeding during tumor resection, the bleeding was easily removed by an aspirator, and the field under the microscope could always be kept clean, were recorded as poor blood supply; those who had more bleeding during tumor resection, and the blood in the field under the microscope was difficult to remove completely by using only one set of aspirator, were recorded as rich blood supply. The amount of intraoperative bleeding, which was estimated by neurosurgeon at the end of the surgery, was also recorded to assess the reliability of the grouping.

### MRI data acquisition

**Figure 1:**
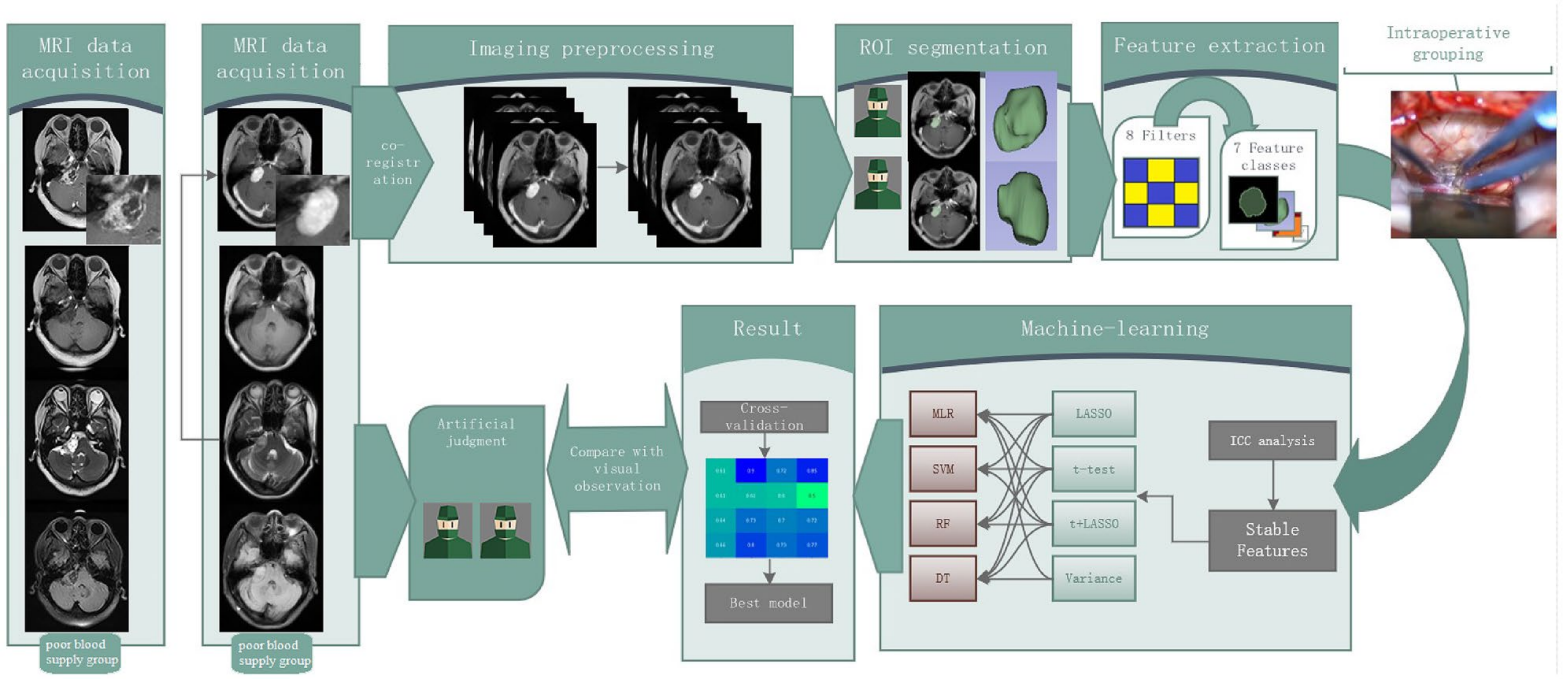
The radiomics workflow in this study. The patient's MRI data was acquired. After uniform preprocessing, two neurosurgeons drew the ROI separately for feature extraction. Samples were grouped by intraoperative recording, the stable features screened by ICC entered the machine learning process to select the best model, which was used to compare the performance with the neurosurgeon's visual observation.

MRI was performed with a MAGNETOM Skyra 3.0 T scanner (Siemens Medical Solutions, Erlangen, Germany) and standard head coil. The imaging sequences in our study included: axial T1-CE (repetition time, 434 ms; echo time, 2.5 ms; slice thickness, 5 mm) with gadopentetate dimeglumine (Magnevist, Bayer Healthcare) was administered by injection through a peripheral venous catheter at a dose of 0.2 mmol/kg, axial T1 data (repetition time, 434 ms; echo time, 2.5 ms; slice thickness, 5 mm), Axial T2 data (repetition time, 434 ms; echo time, 2.5 ms; slice thickness, 5 mm), and axial T2-Flair data (repetition time, 434 ms; echo time, 2.5 ms; slice thickness, 5 mm). Figure 1 shows the radiomics workflow in this study.

### Imaging preprocessing and ROI segmentation

The preprocessing started with the registration (brain) module of the 3D-Slicer program (version 4.11.0, Windows 64bit) to co-registration T1WI, T2WI, and T2-Flair to the T1-CE sequence (Percentage Of Samples 0.002, B-spline Grid Size 14, 10, 12), and the image correction was performed by the N4ITK MRI field bias correction module with (1,1,1) B-spline grid resolution as the confusion matrix. Next, the pyradiomics package was applied in Python environment to perform Image Normalization (normalizeScale = 100), and resampling the image (ResamplePixelSpacing = [3, 3, 3], Interpolator = sitkBSpline).

The region of interest (ROI) was drawn on T1-CE separately by two neurosurgeons with over 5 years of clinical experience using 3D-Slier software. Neurosurgeon A drew all ROIs manually; and Neurosurgeon B applied the Nvidia AI-Assisted Annotation (Nvidia AIAA) segmentation module^12^ to semi-automatically draw ROIs by placing boundary-points and manually corrected misdrawing.

### Radiomic feature extraction

Feature extraction was performed with the python pyradiomics package, using the 8 Filter Classes built into the package, including Original, Wavelet, LoG (sigma [3.0, 5.0]), Square, SquareRoot, Logarithm, Exponential, and Gradient, using 7 feature classes, including First Order Statistics, Shape-based (3D), Shape-based (2D), Glcm: Gray Level Cooccurence Matrix, Glrlm: Gray Level Run Length Matrix, Glszm: Gray Level Size Zone Matrix, Gldm: Gray Level Dependence Matrix, and using the ROI drawn by each of doctors A and B as masks to obtain two sets of features for A and B, respectively.

### Feature selection

The intraclass correlation coefficient (ICC) of feature sets A and B was calculated using the Pingouin package in Python, and only features with high stability (ICC > 0.8) were retained. To minimize human factors, we selected feature set B (ROI drawn by semi-automatic method) to proceed to the next step of feature selection. Feature selection was performed using 4 methods: Student *t* test, Least Absolute Shrinkage and Selection Sperator (LASSO), ANOVA, and *t* test + LASSO. The optimal λ value in LASSO was automatically selected by bootstrap methods.

### Feature classification and model validation

The radiomic features selected by different methods were used to establish the model by the scikit-learn package (Version 0.23.0)^13^ in python by entering them into Multivariable Linear Regression model (MLR), Support Vector Machines (SVM), Random Forest (RF), and Tree Models, respectively. The 5-repeats-3-fold cross-validation method was used, which divided the case samples into the training set and validation set at the ratio of 2:1, and repeated the validation 5 times to obtain a total of 15 predictions results from each, and plotted the receiver operating characteristic curve (ROC) graphs of cross-validation for different feature selection methods and classifier combinations of validation sets respectively, and calculated their average area under curve (AUC). To diagnose potential problems with learning, the learning curve for the best combination is plotted. The best combination was selected by its performance (AUC). The accuracy, sensitivity, and F1-score, which considered both accuracy and sensitivity of the best model, were also calculated.

### Artificial judgment

Artificial judgment was performed by two other neurosurgeons with over 5 years of clinical experience, they were blinded to the intraoperative recording, and MRI image features of both cohorts were identified by visual observation. Higher signal of the tumor on T1-CE sequences, the finding of multiple flow voids on the tumor surface or in the tumor parenchyma, and solid tumors with less cystic, marked the tumor prediction as rich in blood supply. Lower signal of the tumor on T1-CE sequences, no finding of obvious flow voids related to the tumor, and tumors with multiple cystic, marked the tumor prediction as poor in blood supply. The prediction results of two neurosurgeons were recorded and compared with the gold standard (intraoperative record), and the accuracy, sensitivity, and F1-score were calculated and compared with the prediction results of the machine-learning model.

### Statistical analysis

The statistical analysis of baseline data was performed using IBM SPSS Statistics 21. The quantitative data was analyzed using Student’s *t* test, and the qualitative data was analyzed using Pearson’s Chi-square test, p-value < 0.05 was considered as statistical significance.

### Ethics approval

Approval of the Ethical Committee of the Institute was taken for this retrospective study.

## Results

### Baseline data

**Figure 2:**
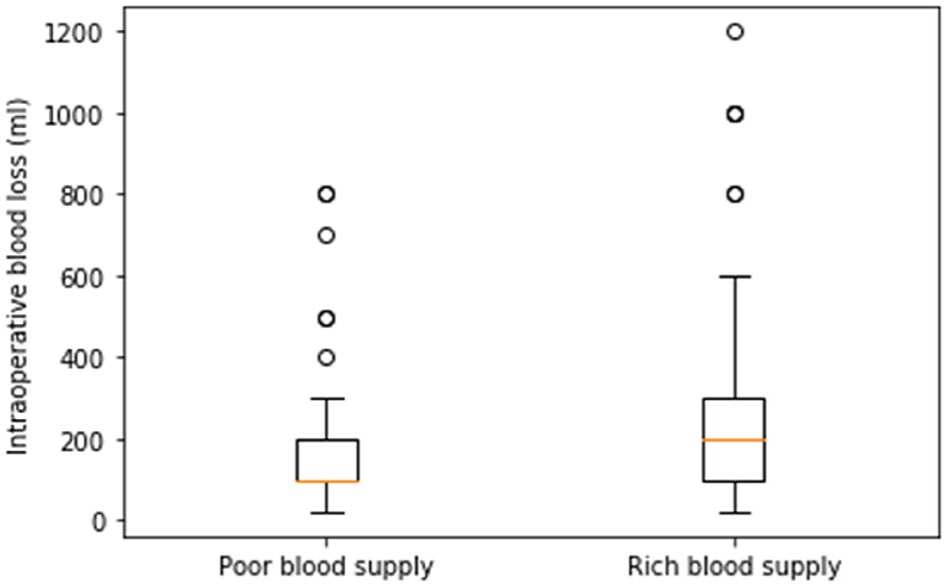
The distribution of intraoperative blood loss in the poor blood supply group and rich blood supply group.

Of 191 patients, there were 109 females and 82 males, aged from 20 to 82 years, with an average age of 50.0 years (SD = 12.1). Referring to the intraoperative records, 119 patients were assigned to the rich blood supply group, and 72 patients were assigned to the poor blood supply group. Patient baseline characteristics between the poor blood supply group and the rich blood supply group are shown in Table 1. The sex and age between the two groups showed no significant statistical difference (p > 0.05). The amount of intraoperative bleeding showed a statistically significant difference (p < 0.05) between the two groups with a mean of 165.3 ml (SD = 156.6) in the poor blood supply group and 251.9 ml (SD = 217.9) in the rich blood supply group. The distribution of intraoperative blood loss in the two groups is shown in Fig. 2.

**Table 1:** Sex, age and intraoperative blood loss distribution between the poor blood supply group and the rich blood supply group.

Characteristics	Groups		Total	p-value
	Poor blood supply	Rich blood supply		
**Sex**				
Male	26	56	72	0.09
Female	46	63	109	
Age (y)	52.7 ± 11.8	48.4 ± 12.0	50.0 ± 12.1	0.73
Intraoperative blood loss (ml)	165.3 ± 156.6	251.9 ± 217.9	219.0 ± 201.2	0.01

Mean ± standard deviation.

### Feature extraction

Each case extracted 1314 features per sequence, and 4 sequences totaled 5256 features. Excluding 10 parametric features per sequence, and computed by filters, we extracted 100 original features per sequence, 172 features in log filter, 688 features in wavelet filter, 86 features in square filter, 86 features in squareroot filter, and 86 features in exponential filter, 86 features in logarithm filter, and 86 features in exponential filter. Computed by image types, we extracted a total of 24 shape features, 757 firstorder features, 984 glcm features, 756 glrlm features, 698 glszm features, and 664 gldm features.

### Feature stability analysis

After the ICCs were calculated, 3918 features with high stability (ICC > 0.8) were retained for the next step of the feature selection process, and features with low stability (ICC < 0.8) were excluded.

### Feature selection and classification

**Figure 3:**
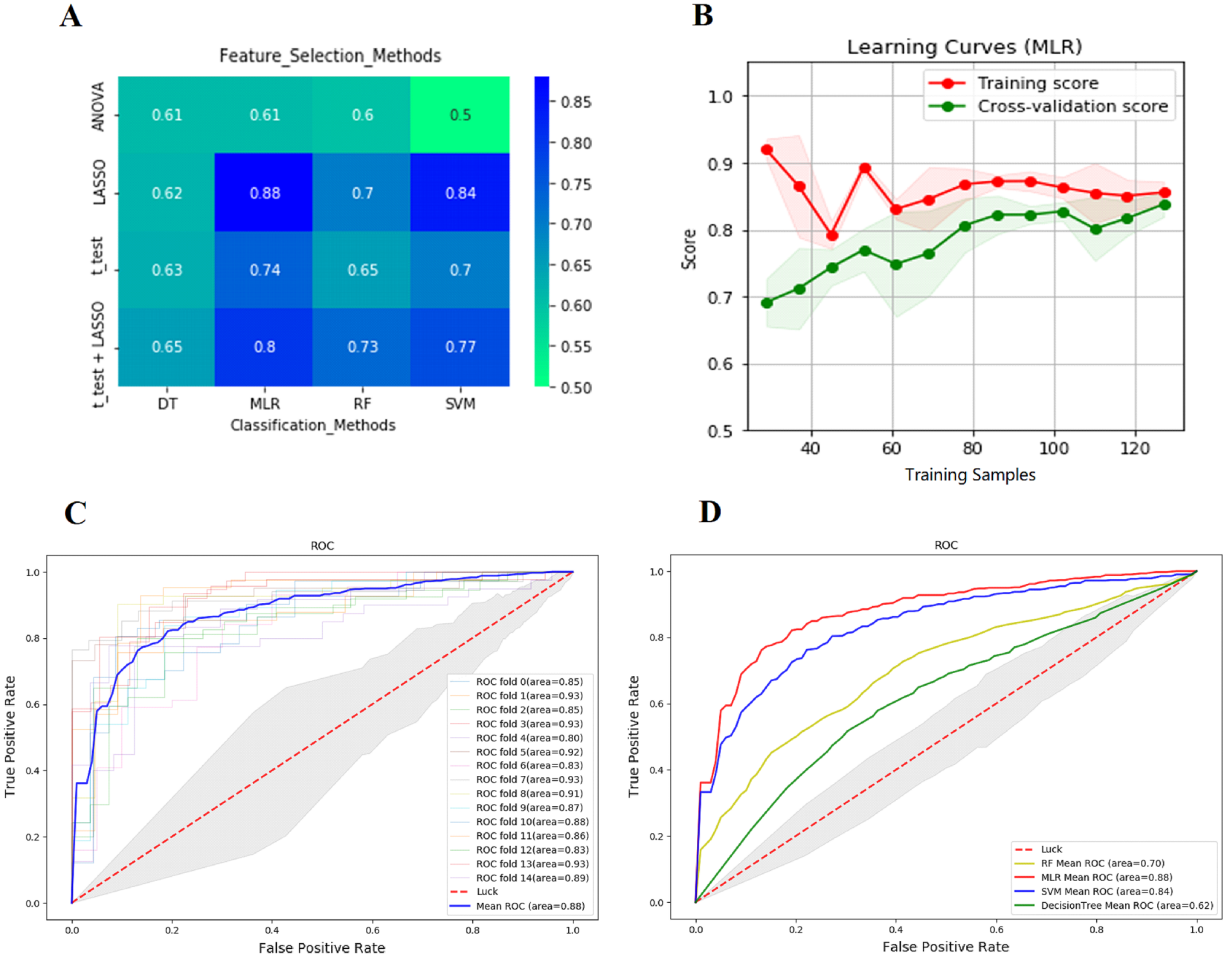
The building of machine-learning models. (**A**) Heatmap of AUC for different feature selection methods and classifier combinations. (**B**) Learning curve of the best combination (LASSO + MLR). (**C**) ROC curves for each of the 15 validations of the LASSO + MLR combination and the average ROC curve for all validation results. (**D**) ROC curves for combinations of LASSO and different classifier.

4 feature selection methods were combined with 4 classifiers, and a total of 16 combinations were applied. Table 2 shows the number of features extracted by different feature selection methods and their average AUC with different classifier combinations. LASSO had the best performance among the 4 feature selection methods, with an average AUC = 0.76 for its extracted features input to the 4 classifiers. The MLR classifier had the best performance among the 4 classifiers, with an average AUC = 0.76 for the 4 feature selection methods input to MLR for prediction. LASSO and MLR were selected as the best combination. The AUCs of all combinations are shown in Fig. 3A and Table 2. The learning curve (Fig. 3B) shows the change in model scores for the training set and cross-validation as the sample size increases. Figure 3C,D show the performance of this combination in 5-repeats-3-fold validations by ROC curves and the difference in the performance of the 4 classifiers when LASSO is used as the feature selection method.

**Table 2:** Number of features extracted by different feature extraction methods and the average AUC.

Feature_Selection_Methods	AUC of classification methods					Number of selected Voxel
	RF	MLR	SVM	DT	Average	
t_test	0.65	0.74	0.70	0.63	0.68	10
LASSO	0.70	0.88	0.84	0.62	0.76	12
ANOVA	0.60	0.61	0.50	0.61	0.58	14
t_test + LASSO	0.73	0.80	0.77	0.65	0.74	12
Average	0.67	0.76	0.70	0.63	0.69

In LASSO feature selection, the optimal λ = 0.02947 was selected from the bootstrap method, and we obtained 41 radiomic features with non-zero coefficients. To reduce the effect of overfitting, following the recommendation of previous studies^14,15^, we kept only 12 features with the largest absolute values of coefficients according to the sample size in the training set divided in this study. The selected features and their coefficient weights in LASSO are reflected in Table 3. In the *t* test + LASSO, because there were too many features that were statistically different at the significant level (α = 0.05 or α = 0.01) in the t-test, we adjusted the significant level (α = 0.0005) and obtained 96 features, and then 25 features were obtained by LASSO selection, and again, we kept only 12 features with the highest absolute values of coefficient weights. In the Student t-test, we retained 10 features that were considered different between the two groups at significant level 0.00003. In the ANOVA, we set the VarianceThreshold to 1e^19^ and obtained 14 features.

**Table 3:** Selected features and their coefficient weights in LASSO feature selection method.

Sequences	Features	Coefficient
T1-CE	T1c_square_glcm_InverseVariance	− 0.050471655
	T1c_square_glcm_JointEnergy	0.033219662
	T1c_squareroot_glszm_SizeZoneNonUniformityNormalized	− 0.051365988
	T1c_wavelet-HHL_firstorder_Kurtosis	0.097581273
	T1c_wavelet-HLL_gldm_DependenceVariance	− 0.055243396
T1WI	T1_wavelet-HLL_glrlm_GrayLevelNonUniformityNormalized	− 0.029010829
T2WI	T2_exponential_glcm_ClusterProminence	0.036160982
	T2_exponential_gldm_DependenceVariance	0.050859759
	T2_exponential_glrlm_ShortRunLowGrayLevelEmphasis	0.064971769
T2-FLAIR	T2_Flair_logarithm_glcm_ClusterShade	0.035088135
	T2_Flair_log-sigma-3-0-mm-3D_firstorder_InterquartileRange	− 0.02945977
	T2_log-sigma-3-0-mm-3D_glrlm_LongRunHighGrayLevelEmphasis	− 0.031169

### Model validation and estimation

The mean of the weighted average accuracy, sensitivity, F1 score, and accuracy in the prediction of tumor blood supply condition by two neurosurgeons in visual observation was 0.67, 0.63, 0.64, and 0.63, respectively. The mean score of MLR in the 3-fold-5-repeats cross-validation of the machine-learning model was 0.787, the mean AUC was 0.88 with a 95% CI from 0.806 to 0.932, and the weighted mean precision, sensitivity, F1-score, and accuracy for predicting the rich blood supply were 0.87, 0.86, 0.83 and 0.83, respectively. Table 4 shows a comparison of the performance results between the two, with the machine-learning model outperforming the visual observation in all aspects of prediction performance.

**Table 4:** Comparison of the performance results between the neurosurgeons in visual observation and machine-learning model.

Reader	Precision	Sensitivity	F1 score	Accuracy
Model	0.87 ± 0.03	0.86 ± 0.02	0.83 ± 0.02	0.83 ± 0.01
**Doctors**				
Doctor A	0.65	0.61	0.62	0.61
Doctor B	0.68	0.64	0.65	0.64
Average	0.67	0.63	0.64	0.63

*F1 score* F-score or F-measure is a measure of a test’s accuracy which is calculated from the precision and recall of the test.

Mean ± standard deviation.

## Discussion

The concept of radiomics was introduced in 2012 to capture the intra-tumoural heterogeneity in a non-invasive way^16^, which provided clinicians with an easily accessible and low-cost means of mining the patient's imaging data for radiomic features that cannot be identified by visual observation, and applied artificial intelligence, machine-learning, or statistical approaches to analyze the acquired high-throughput data to guide clinical practice^17^.

During the surgery procedure, the blood supply of the VS can significantly affect the surgical operation. This is often due to untimely bleeding in hemorrhagic tumors, which makes it difficult for the operator to distinguish important local anatomical structures in the cerebellopontine angle region and makes electrocoagulation more likely to cause damage to adjacent tissue^9^. Some literature suggests a preoperative embolization for tumors with imaging data suggesting a significantly rich blood supply^6,18,19^. Furthermore, in cases with a rich blood supply, spare aspirators should be prepared and care should be taken not to allow raging bleeding to flow into the subarachnoid space, otherwise, the operator will need to devote considerable effort for hemostasis, resulting in the need for unscheduled blood transfusions or extended operation duration. In clinical practice, the degree of blood supply to the tumor is assessed mainly by visual identification of anatomical features on MRI and the degree of enhancement of the tumor on TI-CE. However, the prediction by visual observation of the two neurosurgeons in this study showed that this method was not quite reliable in practice (precision = 0.67, sensitivity = 0.63). Therefore, it is necessary to develop a tool that can more effectively predict the blood supply of VS preoperatively. In this study, we established a reproducible model by combining a multisequence radiomics method with a machine-learning classifier through repeated sampling and cross-validation, which can preoperatively predict the blood supply of VS more accurately. As shown from the comparison in Table 4, the machine-learning model has a significantly better performance than the visual observation in terms of judgment. Our model holds promise for providing surgeons with additional information during preoperative evaluation, such as better assessment of the required operation duration to improve anesthesia; adequate preparation of blood transfusion or autologous blood transfusion, and multiple sets of suction for backup in the operative field preoperatively for tumors with rich blood supply.

**Figure 4:**
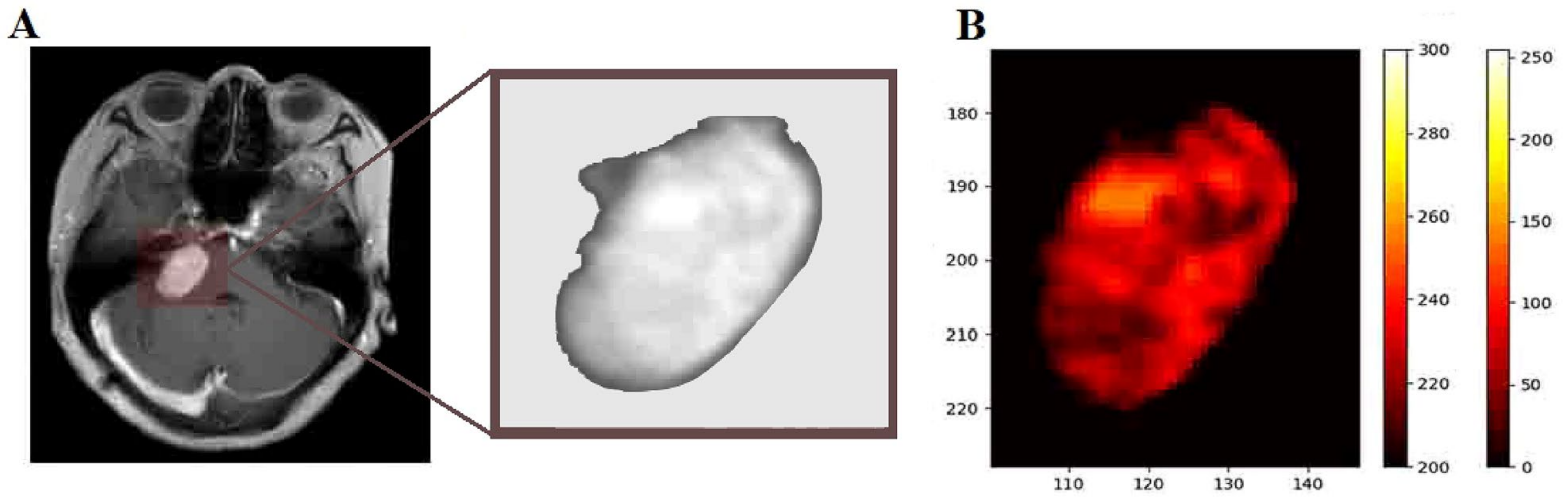
Pseudo-color map generated from ROI of T1-CE sequences. (**A**) T1-CE sequence after imaging preprocessing. (**B**) Pseudo-color map reflects the difference in the details of the grayscale within the ROI, which makes some details more visible.

The main advantage of radiomics than visual observation is that the former is able to extract radiomics features that cannot be identified by the visual observation, so the former can obtain several orders of magnitude more variables that can be used as predictors than the latter. Take the T1-CE sequences of MRI of the poor blood supply group and the rich blood supply group shown on the left side of Fig. 1 as an example, surgeons can distinguish tumors in the poor blood supply group by multiple cystic lesions with low signal through visual observation, while the rich blood supply group is a homogeneous parenchymal tumor with higher signal of the tumor. However, in practice, the MRI of most VS cases is not as typical as the example, and the accuracy of these empirical judgment criteria is not sufficiently reliable. The morphological radiomics features in this study were all excluded in the feature selection, and the extracted radiomics features were all related to the grayscale intensity distribution, which contained certain interpretability. At common resolution contrasts, it is difficult for visual observation to discern subtle differences between grayscale values, and these characteristics are reflected in Fig. 4 in the form of pseudo-color maps, where radiomics downscales the two-dimensional grayscale images, then computes and extracts information from these resulting matrices. For example, the First-Order feature reflects the voxel intensity distribution within the image region defined by ROI; Gray Level Co-occurrence Matrix (GLCM) quantifies gray level dependencies in an image. The detailed explanation is available in the official documentation (https://pyradiomics.readthedocs.io).

In the selection of patient MRI sequences, we included T1-CE as the background for drawing the ROI because most of the VS had significant enhancement on T1-CE^20,21^, and the boundary of the tumor could be clearly outlined. To obtain more voxels, we extracted the T1WI, T2WI, and T2-Flair sequences which were commonly used in the diagnosis of VS, and aligned them to the drawn ROI in T1-CE for co-registration^21,22^. The ADC sequence proved to be valuable in applications such as differential diagnosis, treatment response prediction, and prognostic judgment of VS^23,24^, especially in the determination of cystic and solidity in VS^25^, so we initially included it in the study. However, the co-registration of ADC to T1-CE sequences was always unsatisfactory in terms of anatomical image correspondence, probably due to the distorted and uneven data caused by adjacent structures and inhomogeneous internal structures of the lesion^25^, which may lead to an increase in confounding factors and a potential decrease in reproducibility^26^. Giordano et al. applied multiple ROIs to reduce structural inhomogeneity of the lesion, but the workload of this method was too large, so we finally discarded the ADC sequences.

To obtain a model with better performance, we chose several feature selection methods and classifiers commonly used in the current medical context for combination and finally selected LASSO for feature selection and MLR for modeling based on AUC. As we can see from Table 2 and Fig. 3A, LASSO has significantly higher reliability than other feature filtering methods, and this method has been widely used for high-dimensional data processing^27^, where the coefficients of unimportant features are compressed to zero by a penalty algorithm to achieve dimensionality reduction of the data. The performance of both MLR and SVM classifiers in our results is satisfactory (0.88 vs. 0.84), and both are applicable in medium-sized samples, with MLR demonstrating better performance in this study probably because it usually performs well on binary data.

In machine learning, too many features may lead to the occurrence of overfitting. The recommended ratio of sample size to predictor variables in different studies varies from 20:1 to 10:1, but most recommend a minimum of 10 observations per predictor variable^14,15^. In this study, we followed this principle in order to reduce the possibility of overfitting. As can be seen in Fig. 3B, the scores of the training set and cross-validation approach a satisfactory value and stabilize as the sample size increases, so we believe that there was no significant overfitting occurring in the best combination. The discussion of the transition of radiomics to practical clinical applications has always been a critical issue, and one of the most important aspects of this process is the reproducibility of the acquisition of radiomics features. To reduce the variation among cases in the MRI cases and among different sequences of the same case, we performed the same preprocessing of all the images, including co-registration, correction, normalization, and resampling with same parameters. To minimize human variation, we drew ROIs by two neurosurgeons using different methods and analyzed the extracted radiomics features of both groups for ICC, and excluded those features that differed significantly among groups. However, these basic processes do not necessarily guarantee practical applications. Schwier et al. pointed out that the type of image, pre-processing and ROIs used to evaluate features could significantly change the repeatability of certain features, so the recommended practice was to publish the details of processing and parameter configuration as much as possible^28^. Therefore, on this basis, we published details as much as possible in the whole operation. The yaml file used in the feature extraction process and the MLR model constructed in the feature classification process in this study are available on Supplemental material 1 and Supplemental material 2.

The determination of the actual blood supply classification relied mainly on surgeons’ intraoperative assessment, though subjective errors were inevitable, we standardized the criteria as much as possible within our medical team, and some cases that were difficult to distinguish or whose descriptions were ambiguous when the blood supply was recorded intraoperatively were excluded from this study. We collected and analyzed the intraoperative blood loss of patients, and in our statistics we found that intraoperative bleeding could not be used as a basis for classification of blood supply. However, the results showed a statistical difference between the two groups in terms of intraoperative blood loss when relying on surgeon's intraoperative assessment for grouping, which to some extent reflected the reliability of the grouping. Besides, we collected hemoglobin concentrations from each patient during the perioperative period at 6:00 p.m. preoperatively on the day of surgery and at 6:00 p.m. postoperatively on the day following surgery, and no statistical or practical significance was found in the analysis. Previous studies have shown that methods relying on perioperative blood indicators or calculating the volume of suction fluid are inaccurate for calculating perioperative blood loss^29–31^, which may also support the negative results of our analysis of perioperative hemoglobin concentration changes. A feasible method of quantification is to calculate total intraoperative hemoglobin by measuring the hemoglobin concentration and fluid volume of the suction fluid, and this method is costly but relatively reliable and can be used as a method to further improve related studies.

## Conclusion

The radiomics machine-learning classifiers is an effective method to predict the blood supply of vestibular schwannoma by preoperative MRI data, which has a better performance than neurosurgeons’ judgment by visual observation of MRI image and can provide more information for surgeons to help neurosurgeons make operative strategy.

## Supplementary Information

Supplementary Information 1.

Supplementary Information 2.

## Data Availability

The datasets used or analysed during the current study are available from the corresponding author on reasonable request.

## References

[1] Mahaley M (1990). Analysis of patterns of care of brain tumor patients in the United States: A study of the Brain Tumor Section of the AANS and the CNS and the Commission on Cancer of the ACS. Clin. Neurosurg..

[2] Reznitsky M (2020). The natural history of Vestibular Schwannoma growth-prospective 40-year data from an unselected national cohort. Neuro Oncol..

[3] Halliday J (2018). An update on the diagnosis and treatment of vestibular schwannoma. Expert Rev. Neurother..

[4] Alfaifi A (2018). The top 50 most-cited articles on acoustic neuroma. World Neurosurg..

[5] Yamakami I (2002). Hypervascular vestibular schwannomas. Surg. Neurol..

[6] LeMay DR (1998). Hypervascular acoustic neuroma. Neurol. Res..

[7] Bonneville F (2002). Hypervascular intracisternal acoustic neuroma. J. Neuroradiol..

[8] Teranishi Y (2018). Hypervascular vestibular schwannomas: Clinical characteristics, angiographical classification, and surgical considerations. Oper. Neurosurg..

[9] Talfer S (2010). Surgical treatment of large vestibular schwannomas (stages III and IV). Eur. Ann. Otorhinolaryngol. Head Neck Dis..

[10] Aerts HJ (2016). The potential of radiomic-based phenotyping in precision medicine: A review. JAMA Oncol..

[11] Koos WT (1976). Clinical Microneurosurgery.

[12] Sachidanand Alle, et al. *Nvidia AI-Assisted Annotation *(*AIAA*)* for 3D Slicer* (2019). https://github.com/NVIDIA/ai-assisted-annotation-client.

[13] Abraham A (2014). Machine learning for neuroimaging with scikit-learn. Front. Neuroinform..

[14] Babyak MA (2004). What you see may not be what you get: A brief, nontechnical introduction to overfitting in regression-type models. Psychosom. Med..

[15] Goodfellow I (2016). Deep Learning.

[16] Lambin P (2012). Radiomics: Extracting more information from medical images using advanced feature analysis. Eur. J. Cancer.

[17] Gillies RJ, Kinahan PE, Hricak H (2016). Radiomics: Images are more than pictures, they are data. Radiology.

[18] Perneczky A (1980). Blood supply of acoustic neurinomas. Acta Neurochir..

[19] Rushworth RG, Sorby WA, Smith SF (1984). Acoustic neuroma in a child treated with the aid of preoperative arterial embolization: Case report. J. Neurosurg..

[20] Ahmad TT (2014). Diagnostic accuracy of magnetic resonance imaging in detection of acoustic neuroma. Biomedica.

[21] Robson A (1993). MRI as a single screening procedure for acoustic neuroma: A cost effective protocol. J. R. Soc. Med..

[22] Curati W (1986). MRI in acoustic neuroma: A review of 35 patients. Neuroradiology.

[23] Kunigelis KE (2020). The predictive value of preoperative apparent diffusion coefficient (ADC) for facial nerve outcomes after vestibular schwannoma resection: Clinical study. Acta Neurochir..

[24] Camargo A (2017). Pretreatment ADC values predict response to radiosurgery in vestibular schwannomas. Am. J. Neuroradiol..

[25] Giordano M (2019). Magnetic resonance imaging-apparent diffusion coefficient assessment of vestibular schwannomas: Systematic approach, methodology, and pitfalls. World Neurosurg..

[26] Shiri I (2020). Repeatability of radiomic features in magnetic resonance imaging of glioblastoma: Test–retest and image registration analyses. Med. Phys..

[27] Fonti V, Belitser E (2017). Feature selection using lasso. VU Amsterdam Research Paper in Business Analytics.

[28] Schwier M (2019). Repeatability of multiparametric prostate MRI radiomics features. Sci. Rep..

[29] Johar RS, Smith RP (1993). Assessing gravimetric estimation of intraoperative blood loss. J. Gynecol. Surg..

[30] Guinn NR (2013). Comparison of visually estimated blood loss with direct hemoglobin measurement in multilevel spine surgery. Transfusion.

[31] Stahl DL (2012). Development and validation of a novel tool to estimate peri-operative blood loss. Anaesthesia.

