# Supplementary material for: Prediction of blood supply in vestibular schwannomas using radiomics machine learning classifiers
Source: Sci Rep. 2021 Sep 23;11:18872. doi: 10.1038/s41598-021-97865-5 (PMC8460834; doi:10.1038/s41598-021-97865-5)
Supplement: Supplementary file 1 — Supplementary Information 1. [file 41598_2021_97865_MOESM1_ESM.docx]

# This is an example of settings that can be used as a starting point for analyzing MR data with large(r) (~5mm) slice

# thickness. This is only intended as a starting point and is not likely to be the optimal settings for your dataset.

# Some points in determining better values are added as comments where appropriate

# When adapting and using these settings for an analysis, be sure to add the PyRadiomics version used to allow you to

# easily recreate your extraction at a later timepoint

# ############################# Extracted using PyRadiomics version version ######################################

imageType

Original {}

LoG

# Because of resampling to (3, 3, 3), the use of sigmas 3 mm is not recommended.

sigma [3.0, 5.0]

Wavelet {}

Square {}

SquareRoot {}

Logarithm {}

Exponential {}

featureClass

# redundant Compactness 1, Compactness 2 an Spherical Disproportion features are disabled by default, they can be

# enabled by specifying individual feature names (as is done for glcm) and including them in the list.

shape

firstorder

glcm # Disable SumAverage by specifying all other GLCM features available

- 'Autocorrelation'

- 'JointAverage'

- 'ClusterProminence'

- 'ClusterShade'

- 'ClusterTendency'

- 'Contrast'

- 'Correlation'

- 'DifferenceAverage'

- 'DifferenceEntropy'

- 'DifferenceVariance'

- 'JointEnergy'

- 'JointEntropy'

- 'Imc1'

- 'Imc2'

- 'Idm'

- 'Idmn'

- 'Id'

- 'Idn'

- 'InverseVariance'

- 'MaximumProbability'

- 'SumEntropy'

- 'SumSquares'

glrlm

glszm

gldm

setting

# Normalization

# MR signal is usually relative, with large differences between scanners and vendors. By normalizing the image before

# feature calculation, this confounding effect may be reduced. However, if only one specific scanner is used, or the

# images reflect some absolute world value (e.g. ADC maps, T2maps (NOT T2 weighted)), consider disabling the

# normalization.

normalize True

normalizeScale 100 # This allows you to use more or less the same bin width.

# Resampling

# Increasing the resampled spacing forces PyRadiomics to look at more coarse textures, which may or

# may not increase accuracy and stability of your extracted features. Using a small spacing in large slices generates

# many interpolated voxels, potentially 'masking' the signal contained in the image.

interpolator 'sitkBSpline'

resampledPixelSpacing [3, 3, 3]

# Mask validation

# correctMask and geometryTolerance are not needed, as both image and mask are resampled, if you expect very small

# masks, consider to enable a size constraint by uncommenting settings below

#minimumROIDimensions 2

#minimumROISize 50

# Image discretization

# The ideal number of bins is somewhere in the order of 16-128 bins. A possible way to define a good binwidt is to

# extract firstorderRange from the dataset to analyze, and choose a binwidth so, that rangebinwidth remains approximately

# in this range of bins.

binWidth 5

# first order specific settings

# When normalizing, gray values below the mean will be negative. Shifting by 300 (3 StdDevs 100) ensures that the

# majority of voxels is positive (only outliers 3 SD lower than the mean will be negative).

voxelArrayShift 300

# Misc

# default label value. Labels can also be defined in the call to featureextractor.execute, as a commandline argument,

# or in a column Label in the input csv (batchprocessing)

label 1
